# Latest advances in the regulatory genes of adipocyte thermogenesis

**DOI:** 10.3389/fendo.2023.1250487

**Published:** 2023-08-23

**Authors:** Tao Nie, Jinli Lu, Hua Zhang, Liufeng Mao

**Affiliations:** ^1^ School of Basic Medicine, Hubei University of Arts and Science, Xiangyang, China; ^2^ Scientific Research Center, The First Affiliated Hospital of Guangdong Pharmaceutical University, Guangzhou, China; ^3^ Department of Medical Iconography, The Second Affiliated Hospital, Guangzhou Medical University, Guangzhou, China

**Keywords:** obesity, adipocyte thermogenesis, adrenergic signaling pathway, PKA, UCP1

## Abstract

An energy imbalance cause obesity: more energy intake or less energy expenditure, or both. Obesity could be the origin of many metabolic disorders, such as type 2 diabetes and cardiovascular disease. UCP1 (uncoupling protein1), which is highly and exclusively expressed in the thermogenic adipocytes, including beige and brown adipocytes, can dissipate proton motive force into heat without producing ATP to increase energy expenditure. It is an attractive strategy to combat obesity and its related metabolic disorders by increasing non-shivering adipocyte thermogenesis. Adipocyte thermogenesis has recently been reported to be regulated by several new genes. This work provided novel and potential targets to activate adipocyte thermogenesis and resist obesity, such as secreted proteins ADISSP and EMC10, enzyme SSU72, etc. In this review, we have summarized the latest research on adipocyte thermogenesis regulation to shed more light on this topic.

## 1 Introduction

Obesity is becoming more and more prevalent all over the world and is a huge risk to human health (1). An energy imbalance causes obesity: more energy intake or less energy expenditure, or both. So it is effective to prevent and treat obesity by reducing energy intake or increasing energy expenditure. GLP-1 has been clinically used to treat obesity effectively by decreasing energy intake (2, 3). In these years, lots of studies have reported that non-shivering adipocyte thermogenesis, which always maintains the body temperature, can be promoted to increase energy expenditure and resist obesity. It is a promising strategy to combat obesity by activating the thermogenic adipocytes (4, 5).

**Figure 1:**
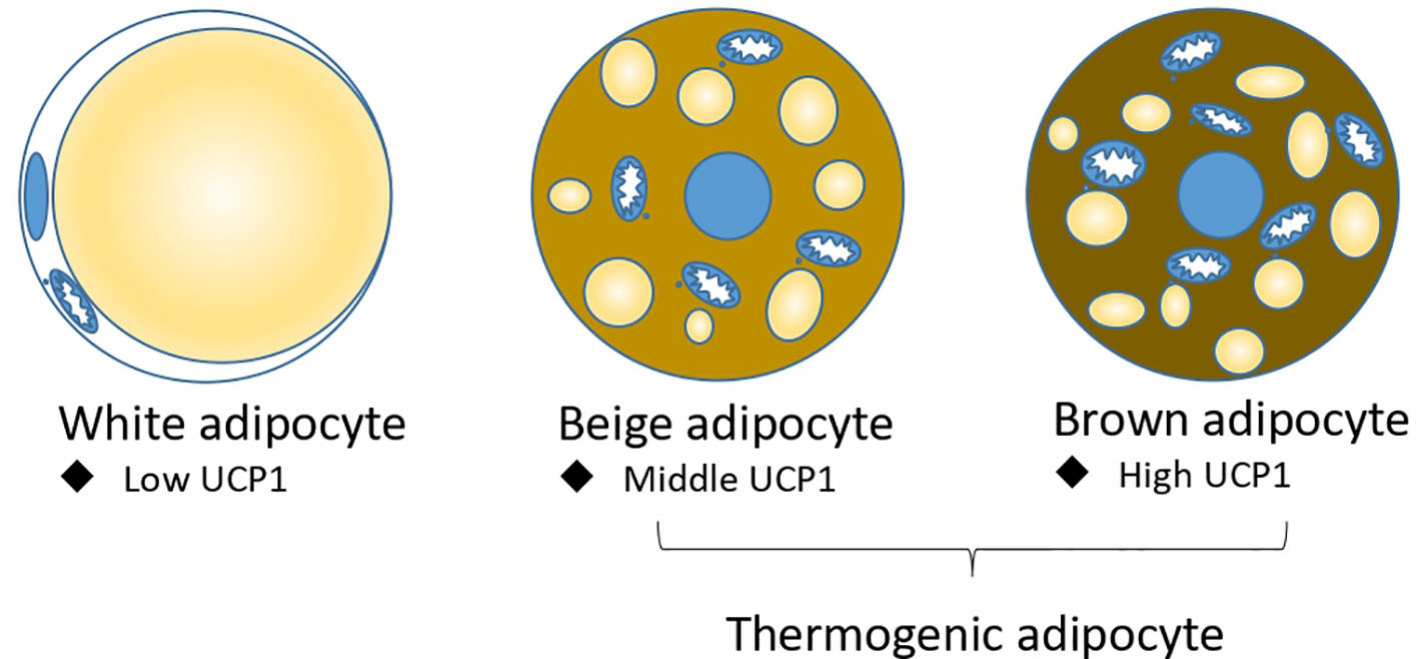
Three types of adipocytes: white adipocyte, beige adipocyte, and brown adipocyte. Beige adipocyte and brown adipocytes are thermogenic adipocytes for non-shivering thermogenesis.

Adipose tissue is classically divided into white adipose tissue (WAT) and brown adipose tissue (BAT). Morphologically, white adipocyte has a unique large lipid droplet, while brown adipocyte has many small lipid droplets and more functional mitochondria (6). Functionally, white adipocyte stores energy and secrets many cytokines, including adiponectin and leptin et ac., to regulate metabolism (7); brown adipocyte highly express a mitochondrial inner membrane protein UCP1 (Uncoupling protein 1), which can dissipate proton motive force into heat without producing ATP. Under cold or pharmacological conditions, a brown-like adipocyte called a beige adipocyte or brite adipocyte, is induced in white adipose tissue (8). Like brown adipocyte, beige adipocyte have many small lipid droplets and highly expressed UCP1 to produce heat; brown and beige adipocytes are also called thermogenic adipocytes (**Figure 1**). Brown and beige adipocytes are functionally the same, but their origins differ. Beige and white adipocytes are mostly *Myf5* negative (9–11), while brown adipocytes are *Myf5* positive and share a precursor cell with myocyte (12). *Prdm16* is the master gene for brown and beige adipocyte identity. Overexpression of *Prdm16* in myoblast can trans-differentiate it into brown adipocyte (13, 14), and the knockout of *Prdm16* in white adipocyte tissue abolished the induction of beige adipocyte by cold (15, 16).

Interestingly, when the beta-adrenergic signaling pathway was ablated in white adipose tissue, a kind of glycolytic beige adipocyte was induced from *Myf5* positive precursor cells (17). Brown adipose tissue is located in the interscapular depot in mammals, and white adipose tissue is mainly located in the subcutaneous and visceral depot (6). It is noted that brown adipose tissue gradually disappeared when humans grew up (18, 19). There are brown and beige adipocytes in the deep human neck (20, 21). In addition, beige and brown adipocytes positively correlate with body weight (22, 23).

**Figure 2:**
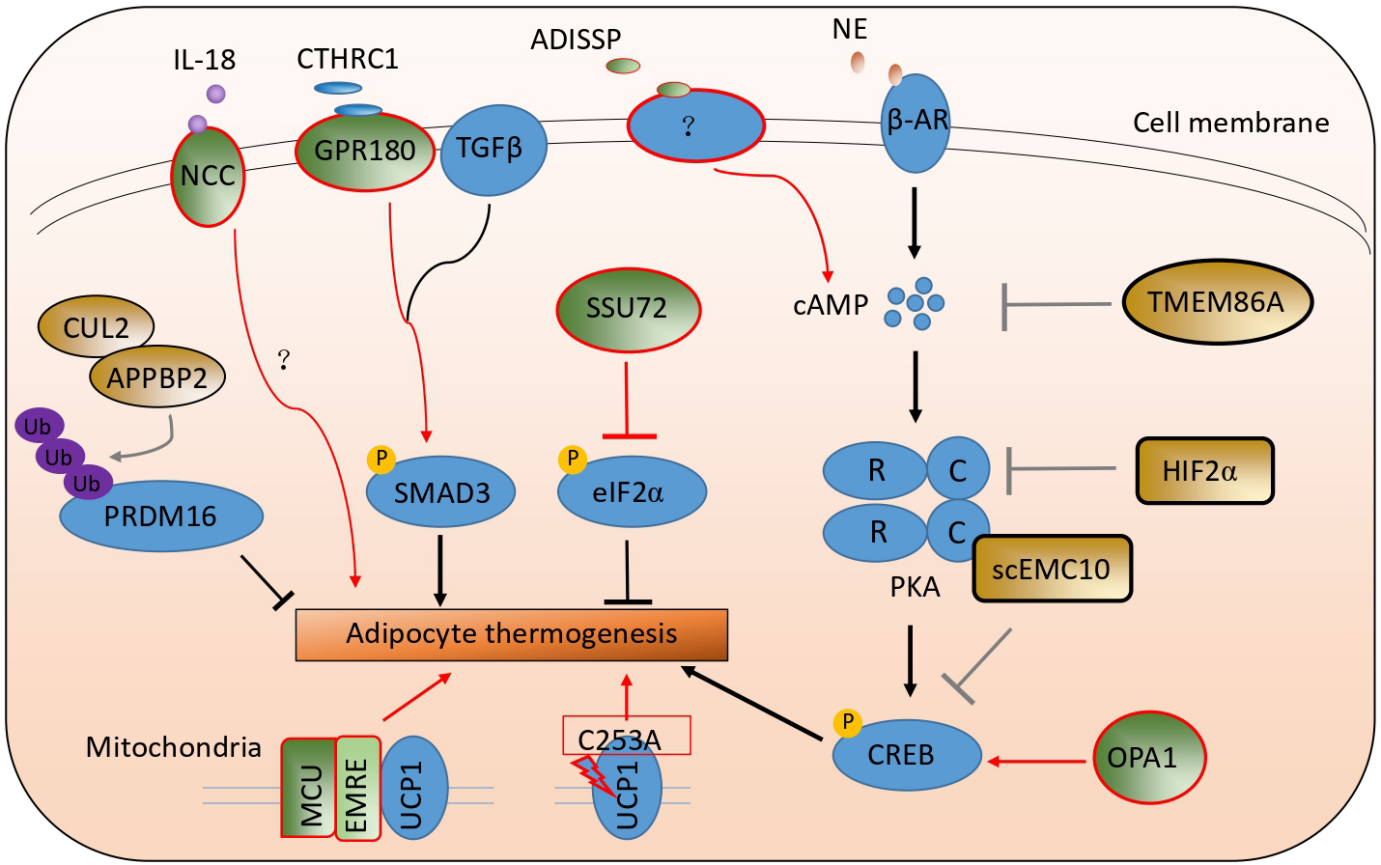
A schematic picture illustrates the latest studies on adipocyte thermogenesis regulation. The green color stands for the positive regulators, and the brown color stands for the negative ones.

Cold exposure is the most effective way to stimulate adipocyte thermogenesis. Upon cold exposure, adipose tissue was sympathetically innervated. The secreted norepinephrine binds with a beta-adrenergic receptor in adipocytes and activates the Gs signaling pathway and adenylyl cyclase to elevate the cycle adenosine (cAMP) contents. And then, cAMP binds with the regulatory subunit of protein kinase A (PKA) to activate its catalytic subunit C to phosphorylate the following transcriptional factor CREB. The phosphorylated protein CREB enters the nucleus and upregulates thermogenic genes, including *Ucp1* et al. (24–27). Many studies have reported that many kinds of cytokines, transcriptional factors, kinases, and membrane receptors, like FGF21, IRF4, and ZFP516 et al., are involved in adipocyte thermogenesis (28–31). Here, we have summarized the latest studies involved in adipocyte thermogenesis listed in **Table 1** and **Figure 2**.

**Table 1:** Genes involved in adipocyte thermogenesis.

Genotype	Targeted tissue	Adipose thermogeneis	Diet	Metabolic symptom	Potential mechanism	Human study	Reference
*Emc10* ^-/-^	Whole body	Up	High fat diet	Decrease body weight and improve insulin sensitivity	Interact with PKA Cα to block its activity	Yes	47
*Adissp* ^Tg^	Adipose tissue	Up	High fat diet	Decrease body weight, increase glycolysis and imporve glucose homeostasis	Activate PKA independent on adrenergic signaling pathway	Yes	32
*Hif2α* ^-/-^	Adipose tissue	Up	Standard chow diet	Enhance white adipose browing upon cold exposeure	Increase the expression of PKA Cα	None	33
*Ovol2* ^boh/boh^	Whole body	Down	Standard chow diet	Increase body weight, cold intolerant and insulisn resistance	Interact with CEBPα to inhibit adipogenesis	Yes	34
*Tmem86a* ^-/-^	Adipose tissue	Up	High fat diet	Decrease body weight, attenuate inflammation and improve insulin sensitivity	Increase cAMP contents	None	35
*Ssu72* ^-/-^	Adipose tissue	Down	Standard chow diet	Dysfucntion of mitochondira and cold intolerance	Inhibit the phosphorylation of eIF2α	Yes	36
*Cul2* ^-/-^ or *Appbp2* ^-/-^	Adipose tissue	Up	High fat diet	Counteracts diet-induced obesity, insulin resistance and dyslipidaemia	CUL2–APPBP2 catalyses the polyubiquitinatio of PRDM16 protein and decreased its half life	Yes	37
*Gpr180* ^-/-^	Whole body	Down	High fat diet	Increase body weight and insulin resistance	Component of TGFβ signaling pathway	Yes	38
*Ncc* ^-/-^	Adipose tissue	Down	High fat diet	Increase body weight and insulin resistance	Mediate IL-18 function	Yes	39
*Opa1* ^Tg^	Whole body	Up	High fat diet	Decrease body weight, resist cold and improve insulin sensitivity	Activate CREB	Yes	40
*Mcu1* ^-/-^ *Emre* ^-/-^	Adipose tissue	Down	High fat diet	Increase body weight and decrease body temperature	Form thermoporter with UCP1	Yes	41
UCP1 C253A	Adipose tissue	Down	High-fat, high-sucrose diet	Cold intolerance, more inflammation in male mice	Increase mitochondria ROS	None	42
*ACE2* ^Tg^	Whole body	Up	Standard chow diet	More theromogenci adipocyte and less adipose tissue weight	Promote the expression of VEGF	Yes	43
*Mt2* ^-/-^	Adipose tissue	Up	High fat diet	Increase sympathetic innervation and energy expenditure	Promote sympathetic neuron induced thermogenesis	Yes	44

## 2 Factors directly act on adipocyte

### 2.1 Secreted proteins

#### 2.1.1 EMC10

Due to differential splicing, endoplasmic reticulum membrane complex subunit 10 has two isoforms: membrane EMC10 and secreted EMC10 (scEMC10) (45, 46). ScEMC10 serum contents in white and Chinese Han human populations were positively correlated with obesity and insulin resistance. After bariatric surgery or exercise and caloric restriction, serum scEMC10 levels decreased. The whole-body *Emc10* knockout mice, displaying more adipocyte thermogenesis and higher whole-body energy expenditure, resisted obesity and insulin resistance induced by high-fat diets. Conversely, *Emc10* overexpression mice were more easily induced into obesity and insulin resistance on high-fat diets, had less adipocyte thermogenesis, and lower whole-body energy expenditure. In addition, the circulating neutralizing antibody to EMC10 also helped mice to reduce body weight gain. Mechanically, secreted EMC10 can be taken up by adipocytes and interacted with the Protein Kinase A catalytic subunit α to inhibit it from phosphorylating CREB. So the decreased phosphorylated CREB proteins led to a reduction in adipocyte thermogenesis (47).

#### 2.1.2 ADISSP

ADISSP (Adipose secreted signaling protein) is a new uncharacterized adipokine, highly and selectively expressed in brown adipose tissue in humans and mice. The expression of this protein is higher in adipose tissue in normal people than in obese people and is negatively correlated with body weight index. The overexpression of *Adissp* in adipose tissue by *Ap2* promoter-driven promoted adipocyte thermogenesis, augmented oxygen consumption, increased glycolysis, elevated body temperature, and resisted body weight gain induced by high-fat diets. In contrast, adipose-specific *Adissp* knockout mice had lower body temperature, less adipocyte thermogenesis, and were more susceptible to high-fat diet-induced obesity and hyperglycemia. ADISSP promoted white adipocyte browning and brown adipocyte activity by paracrine signaling but not by endocrine signaling. A more sensitive assay is lacking to measure ADISSP endogenous circulating contents. The PKA signaling pathway can be activated independently of the beta-adrenergic signaling pathway by ADISSP (32). Though a surface receptor in adipocytes binds to ADISSP, the receptor gene is still not confirmed and unknown.

### 2.2 Transcriptional factors

#### 2.2.1 HIFα

HIFα (Hypoxia-inducible factor α, HIFα) has two family members: HIF1α and HIF2α (48). These two genes were upregulated in white and brown adipose tissue after cold exposure. The adipose *Hif1α*, or *Hif2α*, or double-specific knockout mice had more beige adipocytes in the inguinal adipose tissue and higher energy expenditure compared to the wild-type mice upon cold exposure or CL316243 (beta-adrenergic agonist) stimulation. By RNA-Seq analysis, *Prkaca*, which encodes PKA catalytic subunit α (PKA Cα), was a highly ranked gene in adipose tissue of *Hif2α* knockout mice. Using in silico analysis, miR-3085-3p, directly regulated by HIF2α, was identified to target the evolutionarily conserved region of 3’ UTR of *Prkaca*. So, adipocyte HIF2α could suppress PKA Cα-mediated thermogenic properties by promoting miR-3085-3p expression (33).

#### 2.2.3 OVOL2

Ovo-like zinc finger 2 (OVOL2) belongs to the Ovo family of zinc-finger transcription factors family and is highly conserved in invertebrates and vertebrates (49). The C57BL/6J mice with Oval2 mutations induced by Nethyl-N-nitrosourea (ENU), named *Oval2* ^boh/boh^, were characterized by increased body weight without affecting food intake under a standard chow diet. The boh mutation is a single nucleotide transition from G to A, causing the substitution of tyrosine for a conserved cysteine, and does not affect the OVOL2 protein’s stability. *Ovol2* knockout is embryonically lethal. The heterozygotes with the boh allele and the null allele of *Ovol2* (*Ovol2* ^boh/-^) exhibited overall stronger phenotypes: obesity, reduced energy expenditure, and adipocyte thermogenesis. Overexpression of *Ovol2* in adipocytes reduced total body and liver fat and improved insulin sensitivity in mice fed on a high-fat diet. *Ovol2* is highly expressed in white adipose stromal cells but not in mature white adipocytes. OVOL2 can block adipogenesis by interacting with the C-terminal portion of C/EBPα to inhibit its adipogenic function in both mouse and human adipocytes (34). The mechanism of OVOL2 in brown adipocytes remains elusive and needs more investigation.

### 2.3 Enzyme

#### 2.3.1 TMEM86A

Transmembrane protein 86A (TMEM86A) is a putative lysoplasmalogenase, a close homolog of TMEM86B, and a YhhN family protein member (50, 51). Tmem86a expression was significantly upregulated in adipose tissue on high-fat diets compared to the standard chow diet and heavily enriched in mature adipocytes. Transcriptome-profiling (GEO: GSE94753) also shows that TMEM86A expression is upregulated in abdominal subcutaneous WAT from female patients with obesity compared to individuals without obesity. The adipose tissue-specific *Tmem86a* knockout mice had increased mitochondrial metabolism, adipocyte thermogenesis, and energy expenditure and exhibited significantly lower body weight gain. Mechanistically, the untargeted lipidomics analysis suggested that *Tmem86a* overexpression downregulated the content of lysoplasmalogens, including plasmatic lysophosphatidylethanolamine 18:0 (LPE P-18:0) and adipocyte-specific *Tmem86a* knockout (AKO) increases LPE P-18:0 content in adipose tissue. LPE P-18:0 could reduce the activity of PDE3B (phosphodiesterase 3b), which is abundantly expressed in adipocytes and can degrade cAMP. Furthermore, LPE P-18:0 treatment reduced body weight and fat mass, increased energy expenditure, and strongly activated the PKA signaling pathway in adipose tissue (35).

#### 2.3.2 SSU72

SSU72 is a dual-specific protein phosphatase and is expressed in a tissue-specific manner. Recent studies have demonstrated that SSU72 plays an important role in controlling the carboxyl-terminal domain (CTD) function of RNA polymerase II (RNAPII) and monitoring the liver (52–54). In adipose tissue, SSU72 phosphatase was highly enriched in brown adipose tissue relative to white adipose tissue. *Ssu72* mRNA and protein levels were increased significantly in BAT and WAT after cold exposure. BAT from adipose-specific *Ssu72* knockout mice appeared pale and showed enlarged mitochondria and disorganized cristae structures compared to WT mice. The knockout mice were much more cold-sensitive and intolerant after cold exposure. The expression of thermogenic genes and fatty acid β-oxidation genes was significantly attenuated in the brown adipose tissue of AKO mice.

When endoplasmic reticulum (ER) homeostasis is disrupted, PKR-like ER-regulated kinase (PERK) is activated to phosphorylate the α subunit of eukaryotic initiation factor 2 (eIF2α) at serine 51 (Ser51). EIF2α phosphorylation represses most of the proteins’ translation to reduce ER stress. Dephosphorylation of eIF2α by its phosphatase GADD34 in the liver can improve insulin sensitivity and reduce hepatosteatosis in mice fed high-fat diets. By RNA-Seq analysis, most PERK-eIF2α target genes were significantly upregulated in AKO BAT. Mechanistically, SSU72 can directly interact with eIF2α to inhibit its phosphorylation and increase the protein translation of mitochondrial oxidative phosphorylation and adipocyte thermogenesis in BAT. Furthermore, metabolic dysfunction in *Ssu72*-abated BAT could return to almost normal after restoring *Ssu72* expression (36).

#### 2.3.3 CUL2-APPBP2

PR domain-containing 16 (PRDM16) is a master gene that controls the biogenesis of brown and beige adipocytes by forming a complex with transcriptional and epigenetic factors (12, 13, 15). PRDM16 is dynamically regulated at the post-translational level. Overexpression of euchromatic histone-lysine N-methyltransferase 1 (EHMT1) or chronic treatment with synthetic ligands of peroxisome proliferator-activated receptor-γ (PPARγ) prolongs PRDM16 protein’s half-life (55, 56). CUL2–APPBP2 complex as the ubiquitin E3 ligase was identified to determine PRDM16 protein stability by catalyzing its polyubiquitination. CUL2 functions as a scaffold protein by interacting with an E2 enzyme, elongation B (ELOB), elongation C (ELOC), and APPBP2 substrate receptor, also found in RING E3 ligase complexes (57, 58). *Cul2* depletion in white adipocytes extended the half-life of PRDM16 protein and significantly increased uncoupled cellular respiration. Overexpression of *Cul2* in adipocytes reduced brown/beige-fat-selective genes’ expression. Consistent with the results of *Cul2* deletion, deletion of *Appbp2* also led to higher PRDM16 protein levels and increased expression of brown/beige-fat-selective genes compared with the control cells. APPBP2 (S561N) variant, associated with lower levels of 2 h postprandial serum glucose and insulin, weakly interacted with PRDM16 protein relative to WT APPBP2. Differentiated primary adipocytes from *Appbp2* mutant mice expressed higher thermogenic gene levels than WT adipocytes. Besides, adipose-specific *Cul2* or *Appbp2* knockout mice expressed higher levels of PRDM16 protein and adipose thermogenesis, displayed significantly higher whole-body energy expenditure, and gained less body weight than controls (37).

### 2.4 Membrane receptor

#### 2.4.1 GPR180

The transforming growth factor β (TGFβ) signaling pathway is complex and associated with various human pathologies (59). By comparing the transcriptome of human supraclavicular BAT (scBAT) and subcutaneous WAT and analyzing the transcriptome of the human multipotent adipose-derived stem (hMADS) cells differentiated into beige and white adipocytes, *Gpr180* was found to be upregulated in brown fat on both tissue and cellular level. The knockdown of *Gpr180* shifted brown and beige adipocytes towards a white-like phenotype. The whole body or inducible adipose tissue knockout of *Gpr180* diminished brown and beige adipocyte function and impaired insulin resistance.

The knockdown of *Gpr180* did not affect cAMP levels and phosphorylation of PKA substrates. However, it reduced the phosphorylation of SMAD3 protein at serine 423 in the matured adipocyte. Besides, TGFβ1-induced phosphorylation of SMAD3 and upregulation of *Ucp1* were attenuated in beige adipocytes without GPR180, which indicates that GPR180 is required for full activation of the TGFβ signaling machinery. Collagen triple helix repeat containing 1 (CTHRC1) was identified as GRP180’s potential ligand. Overexpression of *Cthrc1* in male mice prevented body weight gain and increased energy expenditure during the HFD-induced weight gain. In sum, GPR180 and CTHRC1 are the components of the TGFβ signaling pathway for adipocyte thermogenesis. These components regulate low-grade SMAD3 phosphorylation and control thermogenic adipocyte function, whole-body energy, and glucose homeostasis (38).

#### 2.4.2 NCC

Circulating IL18 level is associated with body weight, insulin resistance, and metabolic syndrome in humans and mice (60). *Il18* ablation in mice led to hyperphagia, obesity, insulin resistance, and decreased energy expenditure. Whole-body deletion of *Il18r* increased body weight and decreased energy expenditure, but white adipocyte browning was enhanced in mice on a chow diet (61, 62). This can be explained by the NaCl co-transporter (NCC) acting as an alternative receptor for IL18’s differential function in adipocyte thermogenesis. A single knockout of *Ncc* or a combined knockout of *Il18r* and *Ncc*, but not a single knockout of *Il18r*, blocked adipocyte thermogenesis. Consistent with this, brown adipocytes from *Ncc* ^−/−^ and *Il18r* ^−/−^ *Ncc* ^−/−^ mice, but not those from *Il18r* ^−/−^ mice, showed decreased levels of IL18 and induced uncoupled respiration.

Furthermore, *Ncc* ^fi/fi^ *Ucp1* ^Cre^ mice gained more body weight, had lower adipocyte thermogenesis, and showed worse glucose intolerance and insulin resistance on high-fat diets. There is no difference in UCP1 positive areas and thermogenic genes’ expression in adipose tissue between *Il18r* ^fi/fi^ *Ucp1* ^Cre^ mice and control mice. However, the concise mechanism of IL18 for *Ucp1* promotion is still unclear. It is only known that it is not dependent on the cAMP signaling pathway in brown adipocytes. Overall, IL18 uses NCC to promote thermogenesis in BAT but uses IL18R to enhance glucose sensitivity in WAT (39).

### 2.5 Other proteins

#### 2.5.1 OPA1

The mitochondrial cristae biogenesis protein optic atrophy 1 (OPA1) was significantly downregulated in the human subcutaneous adipose tissue (SAT) of heavy co-twins by gene expression analysis and correlated with mitochondrial gene expression. *Opa1* ^tg^ mice with ubiquitous mild *Opa1* overexpression were slightly resistant to high-fat diet-induced obesity, glucose intolerance, and hepatic steatosis compared to their littermate controls. Similarly, resistance to obesity was observed in a mouse model of *Opa1* haploinsufficiency (63). *Opa1* ^tg^ mice produce more heat at room temperature by promoting BAT function and WAT browning. *Opa1* facilitates cell-autonomous adipocyte browning by upregulating *Kdm3a*, a member of the Jumanji demethylase. This increases brown and beige thermogenic activity by controlling the H3K9 methylation status of *Adrb1* and *Ucp1* (64, 65). Metabolomic profiling further revealed that fumarate levels produced from the urea cycle derived *Kdm3a*-dependent *Ucp1* induction in adipocytes. Moreover, overexpression of *Opa1* can increase cAMP contents to activate CREB to upregulate the rate-limiting urea cycle enzyme *Cps1* (carbamoyl phosphate synthetase-1) in adipose tissue (40). Although OPA1 may not depend on its pro-mitochondrial fusion role, more research is needed to determine the mechanism by which it activates CREB.

#### 2.5.2 MCU and EMRE

By dissipating energy as heat, UCP1 mediates adaptive thermogenesis. Long-chain fatty acids bind on UCP1 to drive proton leak, while purine nucleotides bind on UCP1 to block this uncoupling process (66, 67). Sulfenylation of UCP1 on Cys253 is essential for acute cold-induced uncoupled respiration, while the lysine succinylation of UCP1 reduces its activity and stability (68). Cytosolic calcium can directly stimulate adenylyl cyclase activity to increase cAMP production and PKA activation to induce thermogenesis (69). Two endoplasmic reticulum-located calcium channels, sarco/endoplasmic reticulum Ca^2+^-ATPase 2b (SERCA2b) and ryanodine receptor 2 (RyR2), are involved in ATP-dependent and UCP1-independent calcium cycling machinery to dissipate energy as heat in a beige adipocyte (70).

The mitochondrial calcium uniporter (MCU) complex, which consists of a pore-forming subunit (MCU) and several regulatory subunits, including essential MCU regulator (EMRE) and mitochondrial calcium uptake 1 (MICU1) et al., is a key regulator of mitochondrial calcium (71). *Mcu* BKO (*Mcu* ^f/f^ with *Ucp1* ^Cre^ mice) and *Emre* BKO (AAV-*Emre* gRNA into BAT of Rosa26-LSL-Cas9 with Adipoq^Cre^) mice are hypothermic. They could not maintain their core body temperatures when challenged with cold exposure. Upon adrenergic stimulation, MCU recruits UCP1 through EMRE to form an MCU-EMRE-UCP1 complex (thermo porter), which increases mitochondrial calcium uptake to accelerate the tricarboxylic acid cycle and supply more protons that promote uncoupled respiration. A mutant EMCU (unable to conduct Ca^2+^) could interact with UCP1 at a similar level as the WT EMCU, decreasing the level of UCP1-dependent respiration. MICU1 is the gatekeeper to prevent Ca^2+^ overload in mitochondria by interacting with MCU. Their interaction markedly decreased upon cold exposure or NE/CL-316,243 treatment. AAV-*Micu1* BKO (AAV-Micu1 gRNA into Rosa26-LSL-Cas9 with Adipoq-Cre) enhanced brown adipocyte uncoupled respiration and energy expenditure when activated by NE. The mice carrying the enforcedly assembled thermo porter gained less body weight, more glucose and insulin tolerance, and increased animal energy expenditure; the opposite metabolic phenotypes were observed in *Mcu* BKO and *Emre* BKO mice (41).

#### 2.5.3 Cysteine 253 to alanine (UCP1 C253A)

Because UCP1 loss causes the depletion of most components of the mitochondrial electron transport chain (ETC) in BAT, the interpretation of phenotypes of *Ucp1* KO mice is bewildering and confusing (72). Thermogenic reactive oxygen species (ROS) could reversibly modify a regulatory site (C253) on UCP1 to elevate UCP1-dependent respiration (67). Based on these findings, a mouse model in which the regulatory C253 site on UCP1 is mutated to an alanine (UCP1 C253A mouse) was generated to examine its role in regulating energy homeostasis and metabolic disease. Quantitative proteomics of BAT from WT, UCP1 KO, and UCP1 C253A mice demonstrated that BAT from UCP1 C253A mice maintained expression of the full mitochondrial metabolic proteins. Both male and female UCP1 C253A mice exhibited significantly lower VO2 consumption, energy expenditure, and VCO2 production in response to cold exposure. Fed on a high-fat, high-sucrose (HFHS) diet, male, and female WT and UCP1 C253A mice gained indistinguishable body weight, and their food intake was identical. Remarkably, male but not female C253A mice exhibited more glucose intolerance than WT mice. The proteomic analysis of adipose tissues from both male and female mice on HFHS diets suggested that UCP1 C253A strongly agonizes WAT inflammation in male but not female mice. The mutation of UCP1 C253A increased mitochondrial protein oxidation and systemic inflammation in male mice since the inflammatory cytokine expression was attenuated upon the supplementation of MitoQ (73), a mitochondria-targeted antioxidant. UCP1 C253A male mice treated with β-estradiol showed the decreased expression of inflammatory cytokines, which were significantly increased in BAT of untreated C253A mice (42).

## 3 Factors on the adipose tissue microenvironment

### 3.1 COVID-19

COVID-19 caused patients adipose atrophy, weight loss, and cachexia by activating adipocyte thermogenesis. A transgenic mouse (*ACE2* ^Tg^) that knocked in human angiotensin-converting enzyme 2 (ACE2) demonstrated progressive weight loss alongside ‘wild-type’ SARS-CoV-2 virus infection. SARS-CoV-2 infection augments adipose thermogenesis and increases thermogenic genes’ expression and UCP1 positive cells by histological and immunohistochemical analysis in BAT, sWAT (subcutaneous WAT) and vWAT (visceral WAT). SARS-CoV-2-infected adipose tissues suffered from hypoxia with high HIF1α expression and contained high levels of VEGF, a main target of HIF1α (74, 75). VEGF is a crucial angiogenic factor that augments adipose tissue browning (76, 77). Anti-VEGF mouse neutralizing antibody largely restored the sWAT and vWAT mass relative to the non-immune IgG (NIIgG)-treated sWAT and vWAT. As seen in mouse models, browning phenotypes of adipose tissues were also observed in SARS-CoV-2-infected Syrian hamsters and human patients who died of severe COVID-19 (43).

### 3.2 Zn

Zn is one of life’s most important essential trace elements and has been associated with insulin resistance and adiposity (78). The low level of plasma Zn is associated with obesity. Its supplementation significantly reduces body weight and plasma cholesterol and triglycerides (79, 80). Cold induces sympathetic innervation, which promotes UCP1 expression and Zn secretion from thermogenic adipocytes. However, Zn did not directly influence thermogenic genes’ expression in primary beige adipocytes. Zn stimulated the length of primary sympathetic neurons’ neurite outgrowth to contribute to thermogenesis. Zn injection induced sympathetic innervation in scWAT and BAT.

On the contrary, local injection of Zn chelator TPEN in scWAT and BAT caused a decrease in sympathetic innervation. Furthermore, 6-hydroxidopamine (6-OHDA), which locally ablates sympathetic fibers in BAT and scWAT of Zn-treated HFD mice, blocked the anti-obesity effect of Zn treatment. MT2, cysteine-rich proteins that bind to Zn with high affinity, in scWAT and BAT are upregulated by high-fat diets. MT2 expression is increased in human scWAT samples from obese individuals and positively correlated with body mass index (BMI). Adipose tissue-specific *Mt2* knockout increased VO_2_, thermogenic genes’ expression, and UCP1 protein levels in scWAT and BAT. At the same time *Mt2* overexpression in BAT and scWAT resulted in decreased sympathetic innervation, VO_2_, and thermogenic gene expression without changes in body weight (44).

## 4 Discussion

Adipocyte thermogenesis is regulated by complex transcriptional factors like PRDM16, PPARγ, EBF2, ect, and has been studied extensively (8, 81). Recently, *Emc10*, *Tmem86a*, *Hif2α*, and *Adissp* have been reported to be involved in adipocyte thermogenesis by regulating the PKA-CREB signaling pathway. This pathway is mainly activated by cold exposure or beta-adrenergic agonists and has been proven to be the most effective way to activate adipocyte thermogenesis. Secreted EMC10 can interact with the Protein Kinase A catalytic subunit α to inhibit it from phosphorylating CREB, HIF2α negatively regulated the expression of *Prkaca*. In contrast, TMEM86A blocked the activation of PKA by reducing the level of cAMP. Conversely, OPA1 can increase cAMP contents to activate the PKA-CREB pathway. In addition, the PKA signaling pathway can be directly activated by ADISSP, but its concise molecular mechanism was not clarified. Thus, regulating the PKA-CREB pathway in adipocyte thermogenesis could still be an important research area. Upon ER stress, PERK is activated to increase eIF2α phosphorylation to repress most of the proteins’ translation. SSU72 can directly interact with eIF2α to inhibit its phosphorylation and increase the protein translation of adipocyte thermogenesis in BAT. Otherwise, the knockdown or knockout of PERK was not tested to examine whether it can promote or inhibit adipocyte thermogenesis in this study. ISR (Integrated stress response) pathway, including PERK, PKR, HRI, and GCN2 is also known to regulate phosphorylation of eIF2α (82), so there are still more studies needed to investigate the regulation of eIF2α phosphorylation in adipocyte thermogenesis.

UCP1 protein is always regarded as an independent uncoupling protein to dissipate proton motive force without producing ATP, a new role of UCP1 in adipocyte thermogenesis has been expanded so that it can form a MCU-EMRE-UCP1 complex to increase mitochondrial calcium uptake to accelerate the tricarboxylic acid cycle and supply more protons that promote uncoupled respiration. It was previously reported that SERCA2b and RYR2 in the endoplasmic reticulum promote UCP1-independent adipocyte thermogenesis by regulating ATP-dependent calcium cycling machinery in beige adipocytes (70). This recent work revealed how Ca^2+^ regulates adipocyte thermogenesis in mitochondria and found that UCP1 can interact with other proteins to exert its effect. Investigating whether more proteins can interact with UCP1 to play roles in adipocyte thermogenesis is intriguing. *Oval2* is reported to promote white adipogenesis and regulate beige and brown adipocyte thermogenesis. It’s unknown whether this gene would affect beige or brown adipogenesis since they all share a similar adipogenic mechanism depending on the activation of PPARγ (83). In addition, there is also an alternative way to promote adipocyte thermogenesis by increasing sympathetic innervation in adipose tissue. COVID-19 infection promoted the angiogenesis of adipose tissue and Zn stimulated the length of primary sympathetic neurons’ neurite outgrowth so that more adipocytes could be innervated for adipose thermogenesis. Although the research works mentioned here are novel and interesting, most of them are firstly reported here and based on a single study from a single research group. We consider that it will be more compelling and reliable if these results could be repeated by other research groups. Moreover, studies including more experimental repetitions are needed, in order to confirm that these genes are also effective in human adipocyte thermogenesis. In **Table 1**, we listed research works that include human studies. These research works have just tested the expression levels or functions of these relevant genes, using experiments performed either *in vitro* or in adipose tissue from healthy and obese human patients. However, they did not include sufficient data to support their associations with human energy expenditure. As such, it is still necessary to demonstrate that these genes are able to *in vivo* regulate human energy expenditure.

It is intriguing to combat human metabolic disorders and obesity by targeting adipocyte thermogenesis. In humans, mild cold exposure, which can activate adrenergic receptor signaling pathways, could increase the rates of glucose, fatty acid uptake, and oxidative metabolism in the brown adipose tissue (84, 85). Moreover, the oral administration of mirabegron (a human β_3_ adrenergic receptor agonist approved for overactive bladder treatment), could activate human brown adipose tissue and increase whole-body energy expenditure (86, 87). Furthermore, a retrospective cohort study concluded that humans with positive BAT had a healthier body fat distribution, with a decreased visceral adipose tissue content and an increased subcutaneous adipose tissue content, as well as improved metabolic symptoms, such as lower blood glucose and lipids, and decreased liver fat accumulation (88). Moreover, another independent retrospective cohort study has also reported an association of human positive BAT with improved cardiometabolic health in terms of dyslipidemia, coronary artery disease, congestive heart failure, and hypertension, especially in overweight or obese individuals (89). However, progress in combating obesity by targeting human thermogenic adipocyte tissue is relatively slow due to issues including ethical reasons. Nevertheless, extensive rodent research has demonstrated the feasibility of combating obesity by targeting thermogenic adipose tissues (90, 91). In sum, though most of these recent findings have been analyzed or verified in human adipocytes or adipose tissue, it is still a long and arduous way to transform these basic studies into a clinic.

## Author contributions

TN, HZ, and LM wrote and revised the manuscript. JL revised the manuscript. All authors contributed to the article and approved the submitted version.
